# Dam Methylation Participates in the Regulation of PmrA/PmrB and RcsC/RcsD/RcsB Two Component Regulatory Systems in *Salmonella enterica* Serovar Enteritidis

**DOI:** 10.1371/journal.pone.0056474

**Published:** 2013-02-13

**Authors:** Sebastián Hernán Sarnacki, María del Rosario Aya Castañeda, Mariángeles Noto Llana, Mónica Nancy Giacomodonato, Miguel Ángel Valvano, María Cristina Cerquetti

**Affiliations:** 1 Instituto de Investigaciones en Microbiología y Parasitología Médica, Universidad de Buenos Aires-Consejo Nacional de Investigaciones Ciencias y Técnicas (IMPaM, UBA-CONICET), Buenos Aires, Argentina; 2 Department of Microbiology and Immunology, Centre for Human Immunology, University of Western Ontario, London, Ontario, Canada; Institut National de la Recherche Agronomique, France

## Abstract

The absence of Dam in *Salmonella enterica* serovar Enteritidis causes a defect in lipopolysaccharide (LPS) pattern associated to a reduced expression of *wzz* gene. Wzz is the chain length regulator of the LPS O-antigen. Here we investigated whether Dam regulates *wzz* gene expression through its two known regulators, PmrA and RcsB. Thus, the expression of *rcsB* and *pmrA* was monitored by quantitative real-time RT-PCR and Western blotting using fusions with 3×FLAG tag in wild type (wt) and *dam* strains of *S.* Enteritidis. Dam regulated the expression of both *rcsB* and *pmrA* genes; nevertheless, the defect in LPS pattern was only related to a diminished expression of RcsB. Interestingly, regulation of *wzz* in serovar Enteritidis differed from that reported earlier for serovar Typhimurium; RcsB induces *wzz* expression in both serovars, whereas PmrA induces *wzz* in *S.* Typhimurium but represses it in serovar Enteritidis. Moreover, we found that in *S.* Enteritidis there is an interaction between both *wzz* regulators: RcsB stimulates the expression of *pmrA* and PmrA represses the expression of *rcsB*. Our results would be an example of differential regulation of orthologous genes expression, providing differences in phenotypic traits between closely related bacterial serovars.

## Introduction

The lipopolysaccharide (LPS) is the most abundant component of outer membrane of Gram negative bacteria which structure is divided in three regions: O-antigen polysaccharide, core oligosaccharide, and lipid A [1]. LPS synthesis is a complex process involving various steps. In particular, O-antigen production and assembly in *Salmonella* occurs by mechanisms that require Wzy (polymerase of the repeating subunits), Wzx (flippase that translocated subunit across the membrane) and Wzz (a chain length determinant) (previously Cld or Rol) [1], [2], [3], [4], [5], [6], [7], [8]. Even though there is a significant amount of information on biochemistry and genetics of the LPS synthesis, the regulatory mechanisms that modulate its production are complex and poorly understood. However, it is known that LPS structure is dynamic, showing changes in response to local microenvironment signal. Many of these signals are detected as stimuli by signal transduction cascades. Usually, these systems are composed by a histidine kinase (HK) (sensor protein) that transmits the signal, through a phosphorylation cascade, to a second component, named response regulator [9], [10], [11], [12], [13], [14], [15]. Often, the response regulator is a transcription factor, thereby the result of its phosphorylation is the activation or repression of gene transcription which product is involved in the adaptation to that given microenvironment. The most important two-component regulatory systems involved in LPS modification are PhoP/PhoQ, PmrA/PmrB and RcsC/RcsD/RcsB. PmrA/PmrB and RcsC/RcsD/RcsB two-component regulatory systems of *Salmonella enterica* serovar Typhimurium (*S.* Typhimurium), each activated by different stimuli, independently promote transcription of the *wzz* gene [16]. The expression of *wzz* is also regulated by PhoP/PhoQ via PhoP-mediated upregulation of PmrD, which binds to the phosphorylated form of PmrA protecting it from dephosphorylation by PmrB [17], [18].

In *Salmonella*, regulation of the long chain distribution of the O-antigen contributes not only to an effective barrier [19] but also affect serum resistance and entry into eukaryotic cells [20], [21], [22], [23], [24]. Furthermore, O-antigen length can also modulate acquired immunity. Indeed, Phalipon and coworkers demonstrated that in *Shigella flexneri* induction of an O-antigen-specific antibody response depends on the length of the polysaccharide chain [25]. Also, *Helicobacter pylori* alters its O-antigen structure expressing O-antigen of high molecular weight in response to acidic pH; an important adaptation that would facilitate colonization of the acidic gastric environment [26].

In gammaproteobacteria the DNA adenine methyltransferase (Dam) introduces a methyl group at the N6 position of the adenine of GATC sequence in the newly synthesized DNA strand after DNA replication, generating methylated DNA [27], [28], [29], [30]. DNA methylation status can affect interactions between DNA and proteins such as RNA polymerase or transcription factors [30] that regulate (activate or repress) gene expression generating a plethora of effects. Thus, Dam mutants of *S. enterica* have shown to have many defects particularly in virulence and they have been proposed as candidate vaccines [31], [32], [33], [34], [35], [36], [37], [38]. We have previously shown that a *dam* null mutant of *S.* Enteritidis presents a reduced expression of *wzz* gene and a defective O-antigen polysaccharide chain length distribution [39].

In this work we study the regulation of *pmrA* and *rcsB* expression by Dam methylation in *S.* Enteritidis. In addition, we found that both *wzz* regulators have a regulatory influence on each other.

## Materials and Methods

### Bacterial strains, plasmids, strain construction, and growth conditions

Bacterial strains and plasmids used are listed in Table 1. *S.* Enteritidis #5694 was kindly given by Dr. Anne Morris Hooke, Miami University; originally from Dr. F. Collins'collection, Trudeau Institute, Saranac Lake, New York. Strains #SS218, #SS219 and #SS220 are *S.* Enteritidis isolates from poultry collected from argentine farms. Wild type strains were used to construct mutant strains listed in Table 1. Gene deletions were performed as described by Datsenko and Wanner [40]. Addition of a DNA fragment encoding 3×FLAG epitope tag at the 3′ end of protein-coding DNA sequences was carried out as previously described using plasmid pSUB11 as a template [41] and oligonucleotides *pmrA*-3×FLAG-5′ and *pmrA*-3×FLAG-3′ for PmrA, and *rcsB*-3×FLAG-5′ and *rcsB*-3×FLAG-3′ for RcsB. The mutagenic primers used are listed in Table 2. *S.* Enteritidis was transformed by electroporation as previously described [42]. Gene deletion and the correct fusion of the ORF with 3×FLAG coding sequence were confirmed by sequencing (Macrogen Inc.), and analyzed with Sequencher (Gene Codes Corporation) and Vector NTI software. Bacteria were grown in Luria-Bertani (LB) broth [43] supplemented, as required, with antibiotics at the following final concentrations: ampicilin, 100 µg/ml; chloramphenicol, 30 µg/ml; kanamycin, 40 µg/ml; and tetracycline, 20 µg/ml. For PmrA and RcsB overproduction experiments bacteria were grown at 37°C in N-minimal medium [44], supplemented with 0.2% (w/v) glucose, 0.1 mg/ml casaminoacids, 2 µg/ml Vitamin B1 and 10 mM MgCl_2_ (high Mg^2+^ concentration) or 10 µM MgCl_2_ 100 µM FeSO_4_ (low Mg^2+^ concentration plus Fe^3+^) [16]. Dam mutants were evaluated, phenotypically, determining the absence of methylated GATC sequences [39]. To confirm *pmrA* deletion and p*pmrA* functionality, the resistance to the antimicrobial peptide Polymyxin B assay was carried out as previously described [45].

**Table 1:** Bacterial strains and plasmids used in this study.

	Relevant characteristic(s)	Reference/Source
**Strain**		
*S. enterica* serovar Enteritidis		
#5694	Wild type	Dr. F. Collins collection
#SS218	Wild type	Poultry isolate
#SS219	Wild type	Poultry isolate
#SS220	Wild type	Poultry isolate
SEΔ*dam*	#5694 Δ*dam*	[39]
SEΔ*rcsB*	#5694 Δ*rcsB*	This work
SEΔ*pmrA*	#5694 Δ*pmrA*	This work
SEΔ*rcsB*Δ*pmrA*	#5694 Δ*rcsB* Δ*pmrA*	This work
SEΔ*wzz*	#5694 Δ*wzz_st_*	[39]
SE*pmrA3*×FLAG	#5694 *pmrA*::3×FLAG, Km^r^	This work
SE*pmrA3*×FLAG Δ*dam*	#5694 *pmrA*::3×FLAG Δ*dam*, Km^r^	This work
SE*rcsB3*×FLAG	#5694 *rcsB*::3×FLAG, Km^r^	This work
SE218Δ*rcsB*	#SS218 Δ*rcsB*	This work
SE218Δ*pmrA*	#SS218 Δ*pmrA*	This work
SE219Δ*rcsB*	#SS219 Δ*rcsB*	This work
SE219ΔΔ*pmrA*	#SS219 Δ*pmrA*	This work
SE220Δ*rcsB*	#SS220 Δ*rcsB*	This work
SE220Δ*pmrA*	#SS220 Δ*pmrA*	This work
SE*rcsB3*×FLAG Δ*dam*	#5694 *rcsB*::3×FLAG Δ*dam*, Km^r^	This work
*Escherichia coli* K-12		
DH5α	F- φ80*lacZ*M15 *endA recA hsdR*(r_K_^−^m_K_^−^) *supE thi gyrA relA Δ(lacZYA-argF) U169*	Laboratory stock
**Plasmids**		
pCP20	*FLP* ^+^, λ*c*I857^+^, λp_R_ Rep^ts^, Amp^r^, Cm^r^	[40]
pIZ833	*E. coli dam* gene, Amp^r^	[69]
pKD3	Template plasmid for mutagenesis, Amp^r^, Cm^r^	[40]
pKD4	Template plasmid for mutagenesis, Amp^r^, Km^r^	[40]
pKD46	γ, β, and *exo* from λ phage, *araC-P_araB_*, Amp^r^	[40]
pSUB11	3×FLAG FRT *ahp* FRT *bla* R6KoriV	[41]
pUC18	High copy number cloning vector, Amp^r^	GenBank/EMBL sequence accession number L09136
p*rcsB*	*rcsB*; Amp^r^ (pUC18 backbone)	This work
p*rcsB*as	*rcsB* cloned in antisense orientation to the P*_lac_* promoter; Amp^r^ (pUC18 backbone)	This work
p*pmrA*	*pmrA*; Amp^r^ (pUC18 backbone)	This work
p*pmrA*as	*pmrA* cloned in antisense orientation to the P*_lac_* promoter; Amp^R^ (pUC18 backbone)	This work

**Table 2:** Oligonucleotides primers used in this study.

Gene Targeted	Primer^a^	Sequence^b^ (5′→3′)
**Gene deletion**		
*dam*	*dam*::Cm (F)	TTCTCCACAGCCGGAGAAGGTGTAATTAGTTAGTCAGCATGTGTGTAGGCTGGAGCTGCTTC
	*dam*::Cm (R)	GGCAATCAAATACTGTTTCATCCGCTTCTCCTTGAGAATTACATATGAATATCCTCCTTAG
*pmrA*	*pmrA*: (F)	GCCGCAGATGATATTCTGCAACCGTGCAGGAGACTAAGCGAATAAGTGTAGGCTGGAGCTGCTTCG
	*pmrA*:: (R)	GAAGGGTCATCGCTCTTCGCTGAAAACGCATCAGGCTCACCATATGAATATCCTCCTTAG
*rcsB*	*rcsB*::Km (F)	CCTACGTCAAAAGCTTGCTGTAGCAAGGTAGCCCAATACAGTGTAGGCTGGAGCTGCTTCG
	*rcsB*::Km (R)	ATAAGCGTAGCGCCATCAGGCTGGGTAACGTAAAAGTGATTTACATATGAATATCCTCCTTAG
**Gene epitope tagging**		
*pmrA*	*pmrA*-3×FLAG-5′	TCGCGGGTTTGGCTACATGCTGGTTGCCACTGAGGAAAGCGACTACAAAGACCATGACGGT
	*pmrA*-3×FLAG-3′	GAAGGGTCATCGCTCTTCGCTGAAAACGCATCAGGCTCACCATATGAATATCCTCCTTAG
*rcsB*	*rcsB*-3×FLAG-5′	CTATCTCTCTTCCGTCACCCTGAGTCCGACAGACAAAGAAGACTACAAAGACCATGACGGT
	*rcsB*-3×FLAG-3′	ATAAGCGTAGCGCCATCAGGCTGGGTAACGTAAAAGTGATCATATGAATATCCTCCTTAG
**Gene cloning**		
*pmrA*	*pmrA*-F	GATC*GAATTC*ATGAAGATACTGATTGTTGAAGACGAC
	*pmrA*-R	GATC*GAATTC*TTAGCTTTCCTCAGTGGCAACC
*rcsB*	*rcsB*-F	GATC*GAATTC*CATGAACAATATGAACGTAATTATTG
	*rcsB*-R	GATC*GAATTC*TTATTCTTTGTCTGTCGGACTC
**Verification of predicted construction**		
*dam*	*Rpe*	TACGACAACCTGAACGGTTG
	*damX*	GCAGCGTGCGGTCAACATG
*pmrA*	*pmrB*	CCTGCTCGAACAATTGGATT
	*yjdB*	AAAAACATGTCCCGATGCTC
*rcsB*	*yojN*	AGAGGTTGTATACTGAGGCGGC
	*rcsC*	CTGGCGGAAGAGAAACAACG
pUC18	down*lacz*18	CGTCAGCGGGTGTTGGCGG
**Real-time PCR**		
16S rRNA gen	q-16s-F	GCCGCAAGGTTAAAACTCAA
	q-16s-R	AAGGCACCAATCCATCTCTG
*rcsB*	q-rcsB-F	ACCGCAGCATTAAGACCATC
	q-rcsB-R	CTCAGGGTGACGGAAGAGAG
*pmrA*	q-pmrA-F	AACCAGCATGTAGCCAAACC
	q-pmrA-R	AACCCTCGACCAACACTCTG
*wzz*	q-wzz-F	CGTCGCTTCGTTCTGTATCA
	q-wzz-R	AGGATGTTACCCAGGACACG

Primers were purchased from Invitrogen Inc. and were designed according to the DNA sequence information available for the *S.* Enteritidis strain (*Salmonella* spp. comparative sequencing blast server BLAST Server Database at www.sanger.ac.uk).

a F, forward primer; R, reverse primer.

b Underlined nucleotides indicate the sequence homologous to pKD3, pKD4 or pSUB11. Underlined and italicized nucleotidic regions indicate the restriction endonuclease enzyme cut sites (*EcoRI*)) incorporated into the primer sequence.

### Molecular cloning of *Salmonella pmrA* and *rcsB* genes

DNA extracted from the parental strains of *S.* Enteritidis was used as template for PCR reaction to amplify *pmrA* and *rcsB* genes. PCR amplification was performed with either *Pwo* polymerase (Roche) (for amplification cloning fragments) or *Taq* polymerase (Qiagen). PCR fragments products were separated in agarose gels, purified using a Gel Extraction kit (Qiagen), and then digested using *Eco*RI restriction enzyme (Roche Diagnostics). Ligation with T4 DNA ligase (Rapid Ligation kit, Roche Diagnostics) into pUC18, also digested with *Eco*RI, and dephosphorylated with shrimp alkaline phosphatase (Roche Diagnostics) was performed. Competent *E. coli* DH5α cells were transformed with the ligation mixture by the calcium chloride protocol [46]. Colonies with a white color phenotype from plates with ampicillin and 0.2% (w/v) X-Gal were pooled and screened by PCR using the primers down*lacz*18 combined with *rcsB*-F and *rcsB*-R for *rcsB*, and *pmrA*-F or *pmrA*-R for *pmrA*. Also, pooled colonies were screened by restriction digestion to preliminary identify the orientation of the inserts (with respect the plasmid promoter on sense or antisense). The integrity of the inserts were confirmed by DNA sequencing (Macrogen Inc.), using the sequencing primer M13 forward and M13 reverse, and the inserts were analyzed with Sequencher (Gene Codes Corporation) and Vector NTI software.

### LPS analysis

LPS was extracted as described by Marolda et al [47]. Briefly, from overnight plate culture, samples were adjusted to OD_600_ of 2.0 in a final volume of 100 µl. Then, samples were suspended in lysis buffer containing proteinase K as described by Hitchcock and Brown [48], followed by hot phenol extraction and a subsequent extraction of the aqueous phase with ether. LPS was resolved by electrophoresis in 14% polyacrylamide gels using a tricine-sodium dodecyl sulfate (SDS) system [49], [50] and visualized by silver staining. Each well was loaded with the same LPS concentration determined by the keto-deoxyoctulosonic (KDO) assay [51]. A densitometry analysis was performed using ImageJ software. The ratio of the relative intensity of the lipid A-core band to the average intensity of the bands corresponding to total O-antigen and core+*n* was calculated by quantifying the pixels in a narrow window across the center of each lane. The densitometric analysis was calibrated by determining the ratio of the relative intensity of the lipid A-core region to the average intensity of the O-antigen bands.

### Reverse transcription-PCR and quantitative real-time PCR

Bacteria were grown at 37°C with agitation to an OD_600_ of 0.6. Cells were lysed, and total RNA was isolated using Trizol reagent (Invitrogen) according to the method described by the manufacturer. Contaminating DNA was digested with RNase-free DNase I (Epicentre Biotechnologies), and the purity of all RNA preparations was confirmed by subjecting them to PCR analysis using primers specific for the gene encoding the 16S rRNA (Table 2). After inactivation of DNase, RNA was used as a template for reverse transcription-PCR. Complementary cDNA was synthesized using random hexamer primers (Invitrogen), deoxynucleoside triphosphates, and Moloney murine leukemia virus M-MLV reverse transcriptase (Invitrogen). Relative quantitative real-time PCR was performed with an appropriate primer set, cDNAs, and Mezcla Real (Biodynamics) that contained nucleotides, polymerase, reaction buffer, and Green dye, using a Rotor-Gene 6000 real-time PCR machine (Corbett Research). The amplification program consisted of an initial incubation for 3 min at 95°C, followed by 40 cycles of 95°C for 20 s, 60°C for 30 s, and 72°C 20 s. The primers used are depicted in Table 2. A no-template control was included for each primer set. Melting curve analysis verified that each reaction contained a single PCR product. For the relative gene expression analysis, a comparative cycle threshold method (ΔΔ*C_T_*) was used [52]. The number of copies of each sample transcript was determined with the aid of the software. Briefly, the amplification efficiencies of the genes of interest and the 16S rRNA gene used for normalization were tested. Then each sample was first normalized for the amount of template added by comparison to the 16S rRNA gene (endogenous control). The normalized values were further normalized using the wild-type sample (calibrator treatment). Hence, the results were expressed relative to the value for the calibrator sample, which was 1. Student's *t* test was used to determine if the differences in retrotranscribed mRNA content observed in different backgrounds were statistically significant.

### Protein extracts and Western blotting analysis

Total protein extracts were prepared from bacterial cultures grown at 37°C in LB medium and harvested at an OD_600_ of 0.6. Cells were pelleted by centrifugation and resuspended with Laemmli buffer [53]. Three independent extractions for each sample were added together to minimize differences in protein recovery from sample to sample. For Western blot assays total proteins were boiled for 5–10 min in Laemmli sample buffer, and each lane was loaded with material from approximately 10^6^ CFU before resolved by 12% SDS-polyacrylamide gel electrophoresis (PAGE) gel. Prestained SDS-PAGE standards (Bio-Rad) were used as molecular weight markers (not shown). The gels were blotted onto a Hybond-P membrane (GE Health-care, Madrid, Spain). Ponceau S red staining was used as loading control before blocking in 5% (w/v) dried skimmed milk in PBS. Finally, 3×FLAG fusion proteins were immunodetected using mouse-monoclonal anti-FLAG M2-peroxidase (HRP) antibodies (1∶5,000; Sigma, St Louis, MO). The reacting bands were detected by enhanced chemiluminescence (ECL) (Luminol, Santa Cruz Biotechnology, Santa Cruz, CA) in an Image Quant 300 cabinet (GE Healthcare) following the manufacturer instructions. Blots were photographed, and the intensity of the signals expressed in arbitrary units was determined by densitometry analysis using the public domain NIH Image J software (http://rsb.info.nih.gov/nihimage/). We randomly selected three different bands from the Ponceau S stained membrane to normalize the intensity of the band of interest. Data were analyzed for statistical significance using a nonparametric Mann-Whitney test.

## Results

### Dam methylation participates in the regulation of *pmrA* and *rcsB* genes

**Figure 1:**
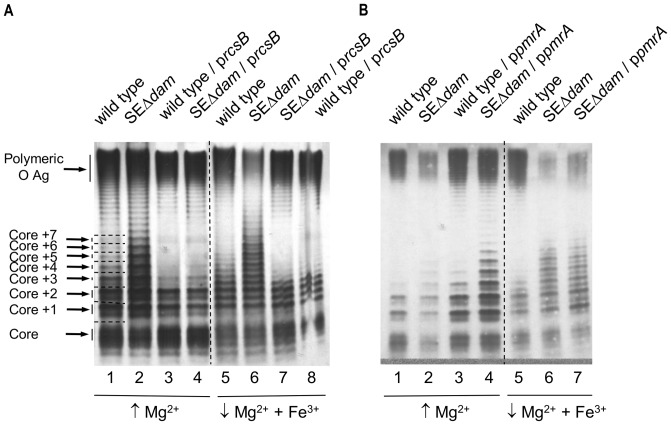
LPS analysis of *S.* Enteritidis strains overexpressing RcsB (A) or PmrA (B) protein. Equal amount of LPS was loaded in each lane and analyzed by Tricine/SDS-PAGE on a 14% (w/v) acrylamide gel followed by silver staining. The concentration of LPS was determined by measuring KDO using the purpald assay. **A.** Lanes 1–4: bacteria grown in N-minimal medium containing 10 mM MgCl_2_; lanes 5–8: bacteria grown in N-minimal medium containing 10 µM MgCl_2_ 100 µM FeSO_4_. **B.** Lanes 1–4: bacteria grown in N-minimal medium containing 10 mM MgCl_2_; lanes 5–7: bacteria grown in N-minimal medium containing 10 µM MgCl_2_ 100 µM FeSO_4_. Plasmids pIZ833, p*rcsB* and p*pmrA* bears the *dam*, *rcsB* and *pmrA* genes respectively.

PmrA and RcsB two-component regulatory system are the only two known *wzz* regulators described in *S.* Typhimurium. To determine whether the LPS phenotype of the *dam* mutant of *S.* Enteritidis (SEΔ*dam*) is related to a diminished expression of these two regulators we analyzed the effect of overproduction of either RcsB or PmrA on the LPS pattern in the *dam* background. Recombinant plasmids containing the *rcsB* and *pmrA* genes cloned into pUC18 were transferred by electroporation in SEΔ*dam* and wild type strains. As we previously described, the LPS pattern of the *dam* mutant showed many more visible bands in the intermediate region of the gel (Fig. 1, lane 2) compared with the banding pattern of the wild-type LPS (Fig. 1, lane 1). The LPS O-antigen profiles of the transformed strains were analyzed in bacteria cultured in LB and under growing conditions known to activate the PmrA/PmrB two-component regulatory system. Results are depicted in Fig. 1. Regardless the culture media used, high Mg^2+^ or low Mg^2+^ + Fe^3+^, we found that RcsB overexpression in SEΔ*dam* mutant (Fig. 1A, lanes 4 and 7) generates an LPS banding pattern comparable to that of the wild type (Fig. 1A, lanes 1 and 5). Similar results were observed when bacteria were cultured in LB medium (not shown). It seems that the presence of high amounts of RcsB in a *dam* background reduces the intermediate region bands observed for SEΔ*dam* mutant (Fig. 1A, lanes 2 and 6). On the contrary, no changes were evident in the LPS pattern of SEΔ*dam* mutant overexpressing PmrA regardless the growth environmental condition, high Mg^2+^ or low Mg^2+^+Fe^3+^ (Fig. 1B, lanes 4 and 7). p*pmrA* plasmid functionality was confirmed by Polymyxin B resistance assay as described in materials and methods (data not shown). Again, similar results for LPS pattern were obtained when bacteria were cultured in LB medium (data not shown). Transformation with plasmids bearing the genes cloned in antisense orientation to the P*_lac_* promoter; (p*pmrA*as, p*rcsB*as), or with empty plasmid vector (pUC18) produced no changes in the O-antigen LPS pattern of any strain studied (data not shown). These data would indicate that the *dam* mutant produces a reduced amount of RcsB protein, suggesting that *rcsB* gene expression is up-regulated by Dam.

**Figure 2:**
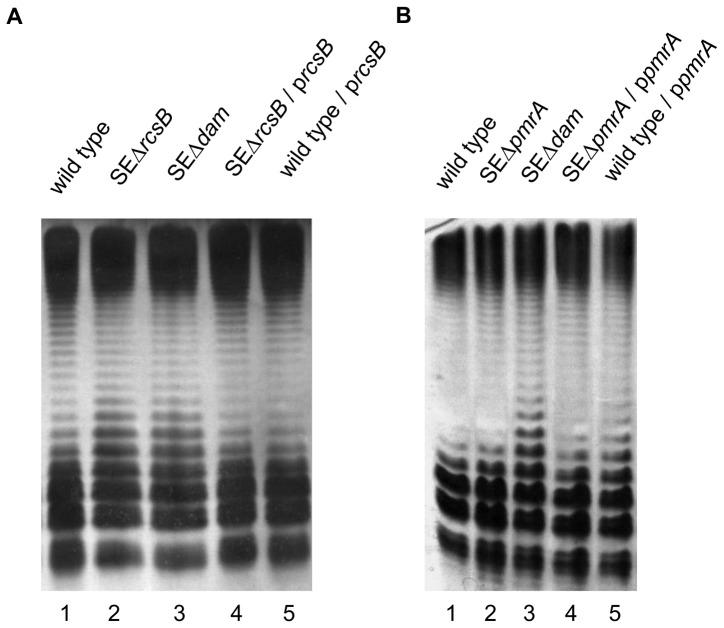
LPS analysis of *rcsB* (A) and *pmrA* (B) mutants of *S.* Enteritidis strains. Equal amount of LPS was loaded in each lane and analyzed by Tricine/SDS-PAGE on a 14% (w/v) acrylamide gel followed by silver staining. The concentration of LPS was determined by measuring KDO using the purpald assay. Plasmids p*rcsB* and p*pmrA* bears the *rcsB* and *pmrA* genes respectively.

Next, we analyzed LPS pattern in the absence of RcsB and PmrA. For this purpose we constructed *rcsB* and *pmrA* deletion mutants of *S.* Enteritidis (SEΔ*rcsB* and SEΔ*pmrA* strains, respectively) using the lambda Red recombination system. As shown in Fig. 2A, the LPS phenotype of SEΔ*rcsB* is similar to that observed in SEΔ*dam* mutant (lanes 2 and 3, respectively). Complementation with the plasmid bearing the *rcsB* gene restored LPS pattern to that found in the wild type strain of *S.* Enteritidis (Fig. 2A). The lack of *pmrA* did not modify LPS pattern in *S.* Enteritidis. As shown in Fig. 2B, deletion mutant SEΔ*pmrA* (lane 2) presents an LPS pattern similar to that of the wild type strain (lane 1). Collectively, these experiments indicate that the reduced *wzz* gene expression observed in SEΔ*dam* mutant correlates with a diminished expression of *rcsB* rather than *pmrA*.

**Figure 3:**
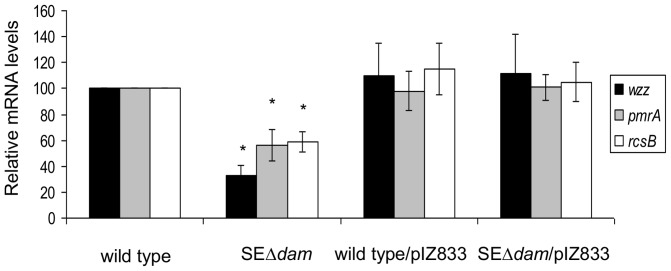
Relative expression of *pmrA rcsB*, and *wzz* mRNA determined by real-time quantitative PCR. Total mRNA was harvested from cultures of SEΔ*dam*, *S.* Enteritidis #5694 (wild type) and complemented strains. The relative mRNA amount was determined by reverse transcription real-time quantitative PCR and related to mRNA levels in wild type strain, set as 1. Values are means ± SD of five independent mRNA extractions performed in triplicates. Plasmid pIZ833 bears the *dam* gen. * significant difference p<0.01 with respect to wild type strain.

**Figure 4:**
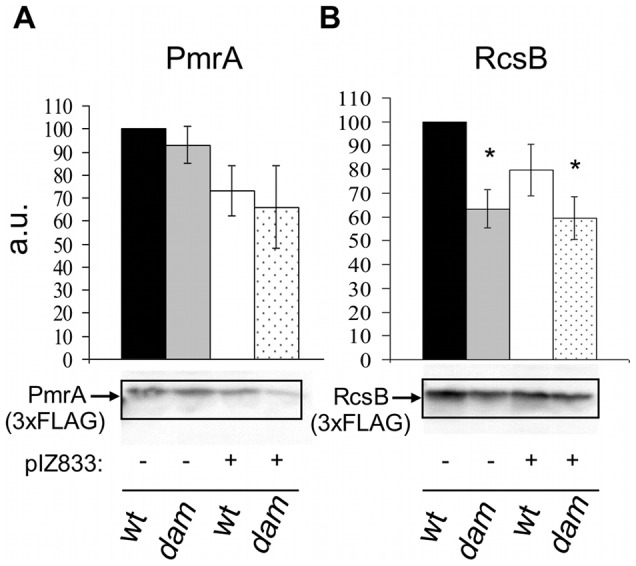
Synthesis of RcsB (A) and PmrA (B) protein in *S.* Enteritidis *dam* mutant. Western blot analysis of total proteins from *S.* Enteritidis #5694 wild type strain and *dam* mutant strains harboring an *rcsB*::3×FLAG (A) or *pmrA::3×*FLAG (B) transcriptional fusion in the chromosome grown in LB medium and harvested at an OD_600_ of 0.6. Protein loading was normalized to 10^6^ CFU. Blots were probed with anti-FLAG antibodies. Band intensity was determined by densitometry; relative intensities are presented in arbitrary units (a.u.). **Panel A.** wt: wild type strain #5694 SE*rcsB*::3×FLAG; *dam*: *dam* mutant strain SE*rcsB*::3×FLAG. **Panel B.** wt: wild type strain #5694 SE*pmrA*::3×FLAG; *dam*: *dam* mutant strain SE*pmrA*::3×FLAG. Plasmid pIZ833 bears the *dam* gene. * Significant difference p<0.05. Data are expressed as means ± SD of percent change in band intensity relative to wild type of five independent experiments performed in duplicates.

In silico analysis has shown the presence of GATC motifs in the coding sequence and/or surrounding nucleotides of *pmrA* and *rcsB* genes [39]. Then we investigated whether Dam methylation regulates the expression of *pmrA*, *rcsB* or both by analyzing the transcription of these genes in the *dam* mutant and the parental strain of *S.* Enteritidis grown to exponential phase in LB medium. By real-time quantitative PCR, the relative expression of both *pmrA* and *rcsB* genes in SEΔ*dam* is reduced (56% and 59%, respectively) compared with the parental strain (Fig. 3). Complementation of *dam* mutation with plasmid pIZ833 restored the expression of *pmrA*, *rcsB* and *wzz* genes to wild type levels (Fig. 3). Thus, a functional Dam results in upregulation of the expression of *pmrA* and *rcsB* genes in *S.* Enteritidis. To analyze whether the reduction in the amount of *pmrA* and *rcsB* mRNA observed in the absence of Dam correlated with the amount of proteins, we quantified PmrA and RcsB in SEΔ*dam* mutant. Because murine anti PmrA or anti RcsB antibodies are not commercially available, we constructed SEΔ*dam* mutants harboring either *pmrA*::3×Flag or *rcsB*::3×Flag transcriptional fusions in the chromosome. Total bacterial proteins were extracted and the relative amount of PmrA and RcsB was determined by Western blot developed with anti-FLAG antibodies (Fig. 4). Densitometry analysis showed that the amount of PmrA produced by the *dam* mutant (as well as the complemented strains) was similar to that produced by the wild type strain (Fig. 4A). On the other hand the relative amount of the RcsB produced by the *dam* mutant was significantly reduced to 63% compared with that of the parental strain (Fig. 4 B).

### RcsB induces the expression of *wzz* and *pmrA*, whereas PmrA represses the expression of *wzz* and *rcsB*

**Figure 5:**
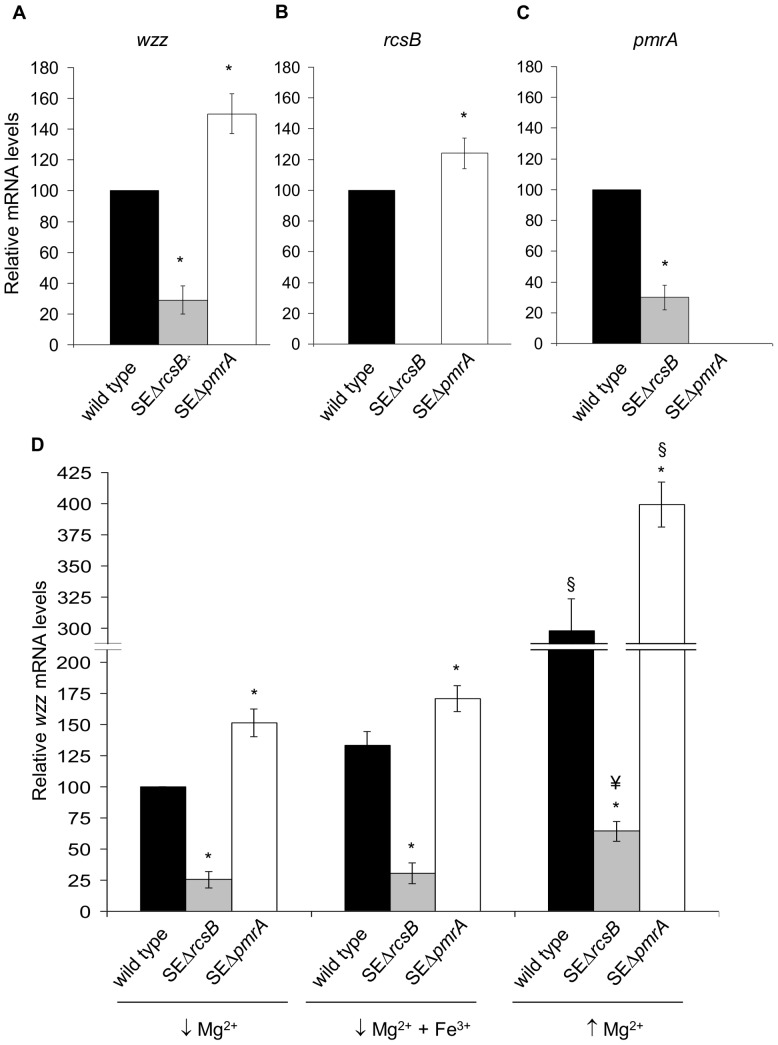
Relative expression of *wzz*, *rcsB* and *pmrA* mRNA in *pmrA* and *rcsB* mutant by real-time quantitative PCR. Total mRNA was harvested from cultures of SEΔ*rcsB*, SEΔ*pmrA* and *S.* Enteritidis wild type #5694 (wild type) grown in LB medium (A,B,C) or grown in low Mg^2+^, low Mg^2+^+Fe^3+^ and high Mg^2+^ (D). The relative amount of *wzz* mRNA was determined by reverse transcription real-time quantitative PCR and related to mRNA levels in wild type strain #5694 (A,B,C) or in wild type strain #5694 grown in low Mg^2+^ (D), set as 1. Values are means ± SD of five independent mRNA extractions performed in triplicates. * significant difference p<0.01 with respect to wild type strain #5694 grown in the same media; § significant difference p<0.01 with respect to the same strain grown in *pmrA-*inducing conditions (low Mg^2+^ and low Mg^2+^+Fe^3+^ ); ¥ significant difference p<0.05 with respect to the same strain grown in *pmrA-*inducing conditions (low Mg^2+^ and low Mg^2+^+Fe^3+^).

Next we analyzed to what extent the expression of *wzz* was reduced in the absence of its two regulators in *S.* Enteritidis. To do that, real-time quantitative PCR was performed using mRNA obtained from knockout *rcsB* and *pmrA* mutants and from wild type strains grown in LB medium. As shown in Fig. 5A, the expression of *wzz* was reduced to 29% in SEΔ*rcsB* mutant compared with the wild type (strain #5694). In contrast, we observed 50% increased expression of *wzz* in SEΔ*pmrA* with respect to the wild type (strain #5694) (Fig. 5A). These features would not be exclusive to wild type strain #5694, since similar results were found using *pmrA* and *rcsB* mutants constructed from clinical isolates of *S.* Enteritidis (data not shown).

These findings prompted us to investigate whether an interaction exists between both *wzz* regulators. Therefore, we determined the expression of *rcsB* in the absence of *pmrA* (SEΔ*pmrA*) and the expression of *pmrA* in the absence of *rcsB* (SEΔ*rcsB*). As shown in Fig. 5B, the expression of *rcsB* in the mutant lacking *pmrA* was increased by 24% with respect to the parental strain cultured in LB medium. In contrast, deficiency in *rcsB* diminished the expression of *pmrA* to 30% compared with the wild type strain grown in the same medium (Fig. 5C). The expression of *rcsB* and *pmrA* was restored in complemented strains (data not shown). To further investigate these interactions, we analyzed *wzz* expression in the wild type, *rcsB* and *pmrA* mutants grown in conditions that stimulate or repress *pmrA*. As shown in Fig. 5D, similar patterns in the expression of *wzz* were found between bacteria cultured under conditions known to activate (low Mg^2+^; low Mg^2+^+Fe^3+^) or repress (high Mg^2+^) *pmrA.* We found that regardless the culture media utilized, *wzz* expression was reduced in SEΔ*rcsB* mutant and increased in SEΔ*pmrA* mutant compared with the parental strain. Interestingly, when the wild type strain was cultured under conditions that repress *pmrA* (high Mg^+2^), the expression of *wzz* was 3 or 2 fold higher compared with the wild type grown in low Mg^+2^ or low Mg^+2^+Fe^3+^, respectively. This increase was even higher in the absence of the *pmrA* gene for any culture medium tested (Fig. 5D). Additional experiments revealed that concurring with the augmented expression of *wzz* (Fig. 5D), the wild type strain increased the expression of *rcsB* and reduced the expression of *pmrA* in high Mg^+2^ compared with low Mg^+2^ (data not shown). These results confirm that *wzz* expression is induced by RcsB and repressed by PmrA. In all cases, the expression of *wzz* was restored in complemented strains (data not shown).

### Is there a third regulator of *wzz* in *S.* Enteritidis?

**Figure 6:**
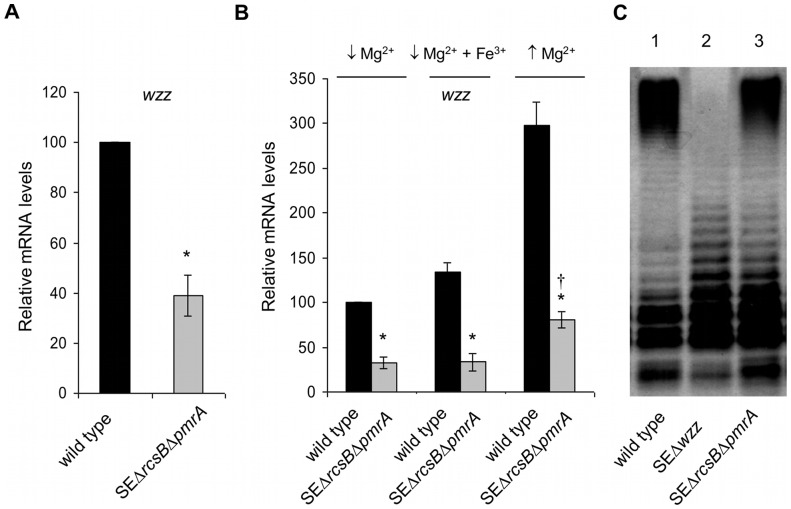
Relative expression of *wzz* mRNA (A and B) and LPS analysis (C) of *rcsB pmrA* double mutant. A,B. Total mRNA was harvested from cultures of SEΔ*rcsB*Δ*pmrA* double mutant and *S.* Enteritidis wild type #5694 grown in LB media (A) or grown in low Mg^2+^, low Mg^2+^+Fe^3+^ and high Mg^2+^ (B). The relative mRNA amount was determined by reverse transcription real-time quantitative PCR and related to mRNA levels in wild type strain (A) or in wild type strain grown in low Mg^2+^ (B), set as 1. Values are means ± SD of five independent mRNA extractions performed in triplicates. * significant difference p<0.01 with respect to wild type strain grown in the same media; † significant difference p<0.05 with respect to same strain grown in *pmrA-*inducing conditions (low Mg^2+^ and low Mg^2+^+Fe^3+^). C. Equal amount of LPS was loaded in each lane and analyzed by Tricine/SDS-PAGE on a 14% (w/v) acrylamide gel followed by silver staining. The concentration of LPS was determined by measuring KDO using the purpald assay.

Results presented in Fig. 5D also show that the expression of *wzz* is induced in the absence of *rcsB* by high Mg^+2^ (*pmrA* repressive condition). This finding is interesting since it suggests the existence of another *wzz* regulator; therefore, we decided to investigate the expression of *wzz* in a double mutant of *S.* Enteritidis lacking *pmrA* and *rcsB* genes (SEΔ*rcsB*Δ*pmrA* strain). As shown in Fig. 6A and B, this double mutant was able to express *wzz* mRNA. We found that regardless the culture condition used the expression of *wzz* was decreased significantly in the double mutant compared with the parental strain. Nevertheless, it is worth noting that for the double mutant the expression of *wzz* was 2.5 fold higher in high Mg^2+^ than in low Mg^2+^ (Fig. 6B). Moreover, LPS analysis showed that -concomitantly with the expression of *wzz*- the double mutant was capable to synthesize O-antigen (Fig. 6C, lane 3). Note that in the absence of Wzz, *S.* Enteritidis (SEΔ*wzz* mutant) is unable to generate O-antigen (Fig. 6B, lane 2). Altogether, our results indicate that, in addition to PmrA and RcsB, another regulator(s) of *wzz* exists in *S.* Enteritidis.

## Discussion

We have reported earlier that the absence of Dam in *S.* Enteritidis causes a defect in the O polysaccharide chain length distribution associated to reduced *wzz* gene expression. Here we investigated whether Dam regulates *wzz* gene expression through its two known regulators, PmrA and RcsB. We found that Dam regulates the expression of both *rcsB* and *pmrA* genes; nevertheless, the *dam* LPS phenotype of *S.* Enteritidis is only associated with RcsB. The fact that SEΔ*dam* mutant exhibits reduced levels of *rcsB* mRNA and a diminished amount of RcsB indicates that the expression of *rcsB* gene is controlled (directly or indirectly) by Dam methylation. The lack (SEΔ*rcsB* mutant) or even a diminished amount (SEΔ*dam* strain) of RcsB resulted in an increased amount of shorter polysaccharide chains similar to the *dam* LPS phenotype. Furthermore, we found that overproduction of RcsB in SEΔ*dam* mutant restores the O-antigen LPS pattern back to that of *S.* Enteritidis wild type. The involvement of RcsB in the regulation of polymerization was reported earlier in *S.* Typhimurium [16]. It was shown that the lack of RcsB affects the mobility in those bands containing 6–10 and 16–22 O-antigen subunits. Unlike serovar Enteritidis, no increase in the amount of shorter polysaccharides was reported for the *rcsB* mutant of *S.* Typhimurium. These subtle differences in the regulation of the O-antigen chain length between two serovars of *Salmonella enterica* would allow them to colonize specific ecological and immunological niches [54].

In *S.* Typhimurium, PmrA not only stimulates *wzz* expression, regulating the O-antigen chain length, but also participates in core and lipid A modifications [16], [55], [56], [57], [58], [59], [60]. Therefore, it would be reasonable to expect a direct participation of PmrA in the O polysaccharide chain length phenotype of *S.* Enteritidis; we found, however, that the absence of PmrA does not cause alterations in the LPS pattern. This is in agreement with the fact that overproduction of PmrA in the *dam* mutant does not restore the defective LPS pattern. On the other hand, our data indicate that Dam methylation (directly or indirectly) does modulate *pmrA* expression. Indeed, *pmrA* mRNA was reduced in the *dam* mutant. This finding is in agreement with microarray analysis data reported by Balbontin *et al.* in *S.* Typhimurium *dam* mutants [31]. Interestingly, despite the diminished amount of *pmrA* mRNA found in the *dam* mutant, PmrA levels remained unchanged. Discrepancies between mRNA transcription and protein translation have been reported earlier [61], [62], [63]. In this regard, different mechanisms related to mRNA stability have been proposed to play a critical role in this phenomenon. Therefore, we conclude that, in *S.* Enteritidis, a functional Dam is required for adequate levels of *pmrA* and *rcsB* gene expression. Also, the diminished amount of RcsB in SEΔ*dam* strain could explain the reduced *wzz* gene expression found earlier in this mutant [39]. We also analyzed the individual participation of PmrA and RcsB in the expression of *wzz* gene in *S.* Enteritidis. As expected, we found that the relative amount of *wzz* is reduced in *rcsB* mutant, indicating that RcsB induces *wzz* gene expression. Surprisingly, in *pmrA* deletion mutant the amount of *wzz* mRNA was higher than in the wild type, indicating that, unlike RcsB, PmrA represses *wzz* gene expression. This finding could explain the normal LPS phenotype of SEΔ*pmrA* (this mutant would not lack Wzz protein).

In order to investigate a putative regulatory effect between both *wzz* regulators, we determined the expression of *rcsB* in a *pmrA* mutant, and *pmrA* expression in an *rcsB* mutant. We found that both regulators affect each other expression. The relative expression of *pmrA* mRNA decreases in the absence of *rcsB*, whereas in the absence of *pmrA*, the relative amount of *rcsB* mRNA increases. These results would indicate that, under the growth conditions used, RcsB stimulates *pmrA* whereas PmrA represses *rcsB*. Also, these findings could explain the elevated expression of *wzz* found in the *pmrA* mutant; in the absence of PmrA, RcsB is derepressed and therefore *wzz* is induced. Regulatory interactions between two-component regulatory systems, coordinating responses to diverse stimuli, have been described. The mechanisms involved in these regulations include phosphatases interrupting phosphoryl transfer in phosphorelays and transcriptional and post-transcriptional modifications [64], [65], [66], [67], [68]. Then, it is possible that an interaction between both PmrA/PmrB and RcsC/RcsD/RcsB two-component regulatory systems would exist in *S.* Enteritidis. In favor of a direct RcsB-mediated regulation of *pmrA*, alignment analysis revealed a potential RcsB protein binding site in *pmrA* gene of *S.* Enteritidis (see Fig. S1 for the bioinformatics analysis performed). Similar results were obtained when the alignment analysis was performed between the conserved regulatory sequences of PmrA binding sites and a putative PmrA binding motif found in *rcsB* gene (supplemental data). Altogether these results would indicate a direct regulation of PmrA protein on *rcsB* gene and RcsB protein on *pmrA* gene. The balance between the expression and repression of *pmrA* and *rcsB* in response to environmental signals suggests a fine tuning of selective genes required for the adaptation to a specific niche.

The experiments performed using double mutant *rcsB pmrA* of *S.* Enteritidis indicate that *wzz* gene is expressed even in the absence of both regulators. Early studies on serovar Typhimurium showed that in the absence of *rcsB* and *pmrA* genes (both *wzz* inducers), the activity of the *wzz* promoter is barely detected and consequently the O-antigen is not synthesized. In fact, the LPS phenotype of *rcsB pmrA* double mutant of *S.* Typhimurium closely resembles that of a *wzz* mutant [16]. On the contrary, our experiments demonstrate that the LPS pattern of *S.* Enteritidis lacking both *rcsB* and *pmrA* genes (*wzz* inducer and repressor, respectively) does conserve O-antigen. These results indicate that, in *S.* Enteritidis, full expression of *wzz* would not depend exclusively on PmrA and RcsB. Although the *wzz* mRNA amount found in *rcsB pmrA* double mutant could be related to a basal expression of *wzz* (but still enough to allow the synthesis of O-antigen), the induction of *wzz* by high Mg^2+^ observed in *rcsB* mutant as well as in *rcsB pmrA* double mutant strongly support the possibility of a third gene regulating *wzz* expression in *S.* Enteritidis.

**Figure 7:**
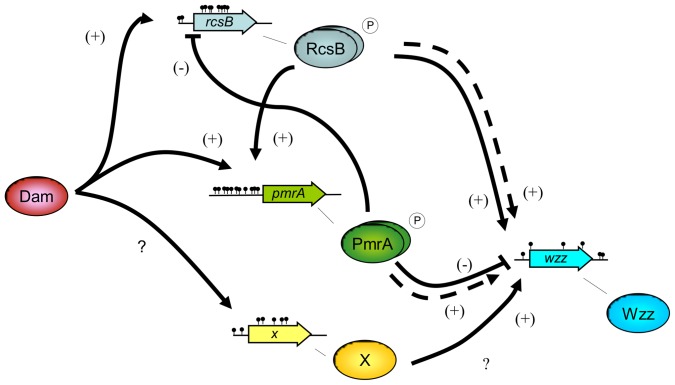
Schematic diagram of the proposed regulatory network of *wzz* gene expression in *S.* Enteritidis. The regulatory cascade for *wzz* gene expression involves Dam methylation, PmrB/PmrA and RcsC/RcsD/RcsB two-component regulatory system and a putative third regulator (X). Proteins are indicated by ovals, whereas genes are symbolized by block arrows. Black dots indicate methylation sites (5′-GATC-3′ sequences). Dashed lines indicate direct interactions demonstrated in *S.* Typhimurium. Positive regulation (induction) is labeled with ↑ and (+), whereas negative regulation (repression) is labeled with ⊥ and (−). The question mark indicates a putative regulation.

In summary, we showed that in *S.* Enteritidis Dam methylation regulates *wzz* expression through *rcsB* and *pmrA* genes; whereas RcsB induces *wzz* gene expression PmrA represses it. We also present evidence that *rcsB* and *pmrA* genes regulate each other; RcsB stimulates the expression of *pmrA* and PmrA represses *rcsB* gene expression. Finally, our results support the existence of a third gene regulating *wzz* expression in *S.* Enteritidis, that can be induced when bacteria is grown in high Mg^2+^. The regulatory network of *wzz* gene expression proposed, including the involvement of the hypothetical third *wzz* regulator, is shown in Fig. 7. Thereby, results presented here would be an example of differential regulation of orthologous genes expression providing differences in phenotypic traits between closely related bacterial serovars.

## Supporting Information

Figure S1 **Bioinformatics analysis.** A. Conserved sequence of PmrA-binding motif. The conserved nucleotides of the sequences corresponding to PmrA binding motif are boxed. B. Molecular analysis of *rcsB* gene region. Diagram of the DNA sequence corresponding to *rcsB* region based on Refseq NC_011294 sequence of *S. enterica* serovar Enteritidis. Alignment analysis performed between the conserved regulatory sequences of PmrA motif and the potential PmrA protein binding site sequences found in *rcsB* gen region are depicted in the correspondent localization. The two know *rcsB* promoters P*rcsB* (located within *rcsD* coding region) and P*rcsDB* (located at −32 pb upstream of the *rcsD* ORF) are marked with arrows. C. Alignment analysis of one of the potential RscB-binding motifs found in *pmrA* gene region with the reported RcsB-dependent regulatory sequences of different enterobacteria. Homologous sequences of the potential RcsB-binding site found in comparison with the reported RcsB motif are in bold. D. Molecular analysis of *pmrA* gene region. Diagram of the DNA sequence corresponding to *pmrA* region based on Refseq NC_011294 sequence of *S. enterica* serovar Enteritidis. Potential RcsB protein binding site sequences found in *pmrA* gen are depicted in the correspondent localization. Next to each potential sequence is indicated the orientation (direct, + or complementary, −), the position relative to the ATG sequence of the gene and the amount of mismatches found in the alignment (mm).
(TIF)
