# Supplementary figures and images for: Dam Methylation Participates in the Regulation of PmrA/PmrB and RcsC/RcsD/RcsB Two Component Regulatory Systems in Salmonella enterica Serovar Enteritidis
Source: PLoS One. 2013 Feb 13;8(2):e56474. doi: 10.1371/journal.pone.0056474 (PMC3572086; doi:10.1371/journal.pone.0056474)

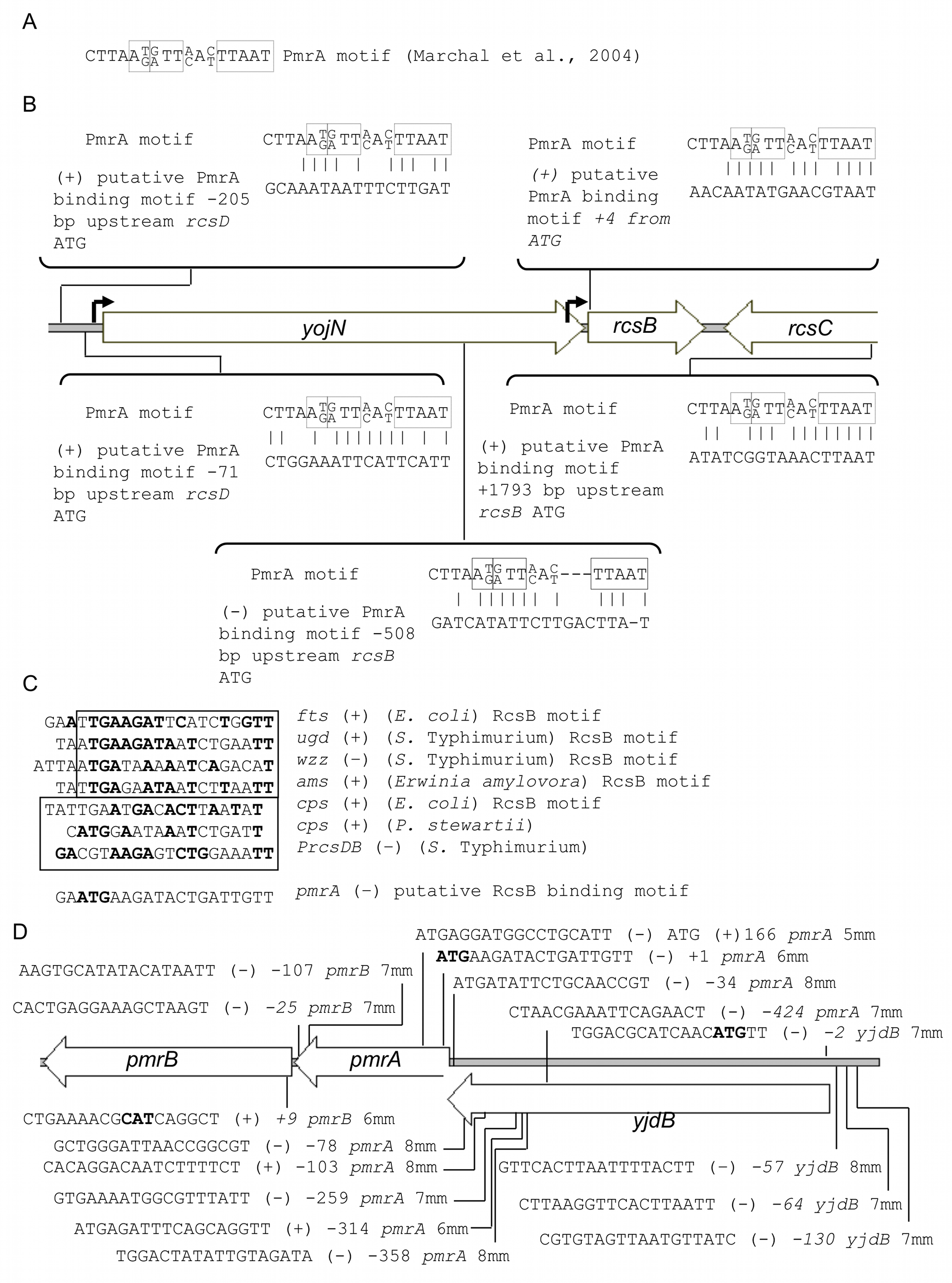

Supplement: Figure S1 — Bioinformatics analysis. A. Conserved sequence of PmrA-binding motif. The conserved nucleotides of the sequences corresponding to PmrA binding motif are boxed. B. Molecular analysis of rcsB gene region. Diagram of the DNA sequence corresponding to rcsB region based on Refseq NC_011294 sequence of S. enterica serovar Enteritidis. Alignment analysis performed between the conserved regulatory sequences of PmrA motif and the potential PmrA protein binding site sequences found in rcsB gen region are depicted in the correspondent localization. The two know rcsB promoters PrcsB (located within rcsD coding region) and PrcsDB (located at −32 pb upstream of the rcsD ORF) are marked with arrows. C. Alignment analysis of one of the potential RscB-binding motifs found in pmrA gene region with the reported RcsB-dependent regulatory sequences of different enterobacteria. Homologous sequences of the potential RcsB-binding site found in comparison with the reported RcsB motif are in bold. D. Molecular analysis of pmrA gene region. Diagram of the DNA sequence corresponding to pmrA region based on Refseq NC_011294 sequence of S. enterica serovar Enteritidis. Potential RcsB protein binding site sequences found in pmrA gen are depicted in the correspondent localization. Next to each potential sequence is indicated the orientation (direct, + or complementary, −), the position relative to the ATG sequence of the gene and the amount of mismatches found in the alignment (mm). (TIF) [file pone.0056474.s001.tif]
